# BikeZ-ETH – A Mass-Cycling Trajectory Dataset from a Controlled Experiment

**DOI:** 10.1038/s41597-026-07247-7

**Published:** 2026-04-23

**Authors:** Kevin Riehl, Shaimaa K. El-Baklish, Ying-Chuan Ni, Anastasios Kouvelas, Michail A. Makridis

**Affiliations:** Traffic Engineering Group, Institute for Transport Planning and Systems, ETH Zurich, Stefano-Franscini Platz 5, 8093 Zurich, Switzerland

## Abstract

This dataset contains high-resolution bicycle trajectory data collected during a controlled mass-cycling experiment conducted on a circular test track at ETH Zurich. A total of 28 cyclists were recorded using aerial video over approximately 30 minutes. The experiment systematically varied the number of simultaneous cyclists and the effective lane width to capture a range of traffic density conditions, including free-flow, disturbed flow, and stop-and-go regimes. Bicycle positions were extracted from drone footage using computer vision-based object detection and tracking, followed by state estimation and Kalman filtering to obtain smooth Cartesian trajectories at frame level. The dataset includes raw video recordings, object annotations, and processed trajectories with spatial and temporal attributes. By isolating cyclist interactions from complex road geometry and mixed traffic, the dataset provides a controlled basis for studying bicycle traffic flow, lateral movement, overtaking manoeuvres, and collective dynamics. The dataset is suitable for use in traffic flow analysis, microscopic modelling, and the development and evaluation of bicycle-specific trajectory prediction methods.

## Background & Summary

The promotion of active modes of transport such as cycling and electric micromobility has become a central component of urban mobility strategies worldwide, driven by sustainability targets such as the United Nations Sustainable Development Goals (the UN SDGs), congestion mitigation policies (e.g., European Union initiatives), and public health considerations^1^. Governments and municipalities increasingly invest in cycling infrastructure and reallocate road space to encourage mode shift away from private motorised transport^2^. The number of studies promoting cycling as a transport mode has also been increasing^3,4^. For example, the E-Bike City project at ETH Zurich has shown that large-scale integration of micromobility into urban transport systems has the potential to significantly reduce congestion, increase road space efficiency, and provide a flexible alternative to car travel, particularly for medium-distance trips^5–8^. Beyond environmental benefits, cycling is associated with significant positive health outcomes (such as improved physical fitness, reduced risk of chronic diseases, better mental health and well-being) and social benefits, contributing to more liveable and equitable urban environments and yielding even further economic benefits^9,10^.

While considerable attention in the literature has been devoted to traffic flow characteristics with a focus on motorised vehicles, little is known about the study of bicycles and micromobility^11^. The same problem exists for research related to traffic safety. Every year, road traffic accidents cause approximately 1.24 million fatalities, with vulnerable road users (VRUs) – and in particular cyclists – accounting for the largest share of these deaths^12^. Despite the rising number of severe traffic accidents involving bicycles and VRUs^13,14^, research on traffic safety and the enhancement of advanced driver assistance systems and safety technologies remains strongly under-represented in the literature, which continues to emphasise pedestrians and motorised vehicles^15,16^.

High-resolution trajectories allow us to model traffic flow dynamics in networks to better understand congestion formation, lateral movement interaction effects, and phenomena like the propagation of stop-and-go waves, oscillations, hysteresis, and capacity drop – which cannot be observed in such a detailed manner when compared with aggregate traffic measurements. Moreover, trajectories can be used to model microscopic driving behaviours, such as car-following & lane-changing manoeuvres for motorised vehicles^17–20^. Furthermore, data-driven trajectory forecasting is a promising branch of research as it helps to improve the safety functions of advanced driver assistance systems and autonomous driving algorithms. By anticipating the potential next steps of a pedestrian or bicyclist, motorised vehicles (especially buses and trucks) could proactively react to avoid collisions and accidents^21,22^.

For bicycles, trajectory data are particularly important due to non-lane-based movement, heterogeneous riding strategies, and strong interactions driven by both physical and social factors^23^. Improving the understanding of cyclist behaviour and interactions is therefore essential not only for infrastructure planning and traffic flow theory, but also for the development of safety-oriented technologies. Advanced driver assistance systems and autonomous driving systems rely increasingly on accurate behavioural models and trajectory forecasting of surrounding road users; insufficient representation of cyclist dynamics can lead to suboptimal or unsafe system behaviour, especially in mixed traffic environments^15,16,24,25^. However, the availability of publicly accessible bicycle trajectory datasets remains scarce, limiting reproducibility and comparative evaluation across studies.

Several bicycle trajectory datasets and experimental studies have been published in recent years, yet each exhibits important limitations. Early datasets were often restricted to small sample sizes or short observation durations, limiting their ability to capture collective dynamics and long-term behavioural patterns^26^. Other datasets focused on complex urban intersections in China and Europe, where interactions with motor vehicles, pedestrians, and geometric constraints complicate the isolation of cyclist-cyclist interactions^27–29^. Large-scale benchmarks such as the Tsinghua-Daimler dataset^30^ or EuroCity Persons^31^ provide valuable visual data but lack high-resolution and continuous bicycle trajectories in a consistent Cartesian coordinate system. The Stanford Drone Dataset offers high-quality annotations and trajectories^32^, yet the presence of mixed traffic, obstacles, and heterogeneous environments makes it difficult to study bicycle traffic flow in a controlled manner.

In addition to observational datasets, several dedicated mass-cycling experiments have been conducted^33–38^. While these experiments provided valuable insights into bicycle flow dynamics, many were limited to single-file dynamics^33–35^ or did not result in publicly available trajectory datasets^36–38^. As a result, opportunities for reuse, benchmarking, and comparative analysis remain limited.

Naturalistic traffic data, capturing real-world behaviour under realistic conditions, is essential for the development of robust traffic flow models and safety-critical systems. Such data cannot be generated in simulations or laboratory settings, as these environments lack the complexity and variability of real-world traffic. Traditional methods for collecting bicycle movement data, including stationary cameras and loop detectors, suffer from significant limitations. Fixed cameras are constrained by limited spatial coverage and occlusions, particularly in urban environments with complex geometries^28,36,39–42^. Loop detectors capture only aggregate measurements at discrete locations and cannot resolve individual trajectories or interactions^43,44^.

Unmanned aerial vehicles (drones) overcome many of these limitations by providing a stable, top-down perspective with high spatial and temporal resolution. Drones allow flexible deployment over complex road geometries and enable the recording of continuous trajectories with minimal interference to traffic participants. This measurement approach has proven particularly effective for capturing detailed interactions, acceleration and deceleration manoeuvres, and transitions between free-flow and congested states^19,45,46^. For bicycle traffic, aerial recordings are especially advantageous, as they avoid occlusions caused by close rider spacing and allow precise estimation of lateral movement and spacing behaviour. The TUMDOT-MUC dataset is known to be the first naturalistic open dataset containing a large number of bicycle trajectories collected using drones^47,48^. However, only limited congested traffic states were observed in that particular real-world environment.

Motivated by these considerations, we conducted a controlled, real-world, closed-loop, mass-cycling experiment on a circular test track at the Hönggerberg Campus of ETH Zurich. The experiment involved recordings lasting around 30 minutes (in total more than 16,054 bicycle-frame observations), 28 unique cyclists, and systematically varied density contexts through variation of the number of simultaneously riding cyclists and the effective lane width. The experiment was designed with four goals in mind: (i) to observe mass-cycling traffic dynamics in a controlled setup, (ii) to record very long bicycle trajectories for individual cyclists, which requires a closed-loop track, (iii) to produce the complete fundamental diagram (FD) of bicycle flow^11^ and collect behavioural parameters to calibrate microscopic simulation models, and (iv) to observe interactions between bicyclists without effects of road geometry or mixed traffic. Aerial video recordings were collected using a drone-based platform, and individual bicycle trajectories were extracted at frame level using computer vision-based tracking, state estimation, and extended Kalman filtering methods^46^. The resulting dataset “BikeZ-ETH-RA” (*RA* for round about) provides long, continuous trajectories of individual cyclists under a wide range of traffic states, enabling detailed analysis of bicycle traffic flow, cyclist interactions, and behavioural variability in a reproducible real-world setting. In contrast to the previous datasets, our controlled experiment design allowed us to exclude interference from road-contextual properties, to study physical and social dynamics only, and to observe very long trajectories of the same bicycles, rather than many short trajectories from cyclists entering and leaving the scene. This makes it particularly interesting for study, e.g., in trajectory prediction research^22^.

### Comparison with previous cycling experiments

Compared to previous cycling experiments, the presented experiment here offers several distinctive advantages. First, it combines a fully controlled experimental design with real-world conditions. While earlier controlled experiments focused primarily on single-file dynamics, short observation windows, or all bicycles following each other on one line^33–35^, the closed-loop circular track used in this study enabled the recording of long, continuous trajectories of individual cyclists over extended periods, while allowing the observation of overtaking and other interactions. This is essential for analysing steady-state behaviour, transient phenomena, and repeated interactions among the same cyclists, which are difficult to capture in open or intersection-based settings. Second, the dataset is publicly available and includes both raw aerial video recordings and processed, frame-level bicycle trajectories in a consistent Cartesian coordinate system at a high frequency (25 FPS). Despite the existence of several large-scale cycling experiments^36–38^, most did not release trajectory data, limiting reproducibility and reusability^49^. Table 1 summarises several experiments conducted to collect bicycle trajectories. The availability of long, high-resolution trajectories makes this dataset suitable for benchmarking microscopic traffic models, calibrating simulation frameworks, and developing data-driven methods for bicycle trajectory prediction. By providing controlled, high-quality empirical data on cyclist interactions, the dataset contributes to closing an important gap in the data foundation for bicycle-aware traffic modelling and safety-critical system development.

**Table 1:** Comparison of dedicated bicycle traffic experiments.

Location	Date	Avail.	Particip.	Record.	Track	Lane Width	Context	Controlled	Description
Hefei, China^34^	3 Nov 2009, 7 Nov 2009, 19 Apr 2010	No	—	Fixed camera (high-angle)	Oval (146 m)	0.8 m	Bicycle-only	Yes	Single-file following behaviour under varying densities
Wuppertal, Germany^33^	6 May 2012	Yes	30	Fixed camera (high-angle)	Oval (86 m)	0.8 m	Bicycle-only	Yes	Single-file bicycle acceleration and following under varying densities
Weijin Street, Tianjin, China^36^	23 Oct 2015	No	—	Fixed camera (high-angle)	Straight path (15.2 m)	5.8 m	Two-wheelers-only	No	Naturalistic urban cycling behaviour
Anshan West Road, Tianjin, China^36^	23 Oct 2015	No	—	Fixed camera (high-angle)	Straight path (7.8 m)	3.2 m	Two-wheelers-only	No	Naturalistic urban cycling behaviour
Anqing, China^37^	7 May 2016	No	100	Fixed camera (high-angle)	Circular (8–11 m radius)	3.0 m	Bicycle-only	Yes	Free-flow, following, overtaking, and stop-and-go under varying densities
Beijing, China^36^	~ 2017	No	6	Fixed camera (high-angle)	Straight path (75 m)	~6 m	Bicycle-only	Yes	Cyclists’ response to obstacles (ball, cylinder, pedestrian)
Rotterdam, Netherlands^38^	25 Apr 2018	No	178	Fixed camera (top-down)	Elliptical ( ~ 80 m)	Various	Bicycle-only	Yes	Free-flow, following, and merging
Anqing, China^37^	28 Sep 2019	No	100	Fixed camera (high-angle)	Oval (4–7 m radius with 13 m straight segments)	3.0 m	Bicycle-only	Yes	Free-flow, following, overtaking, and stop-and-go under varying densities
Munich, Germany^47,48^	Oct 2022	Yes	34	Drone (top-down)	Straight path with traffic signals (140 m)	1.35–2.90 m	Two-wheelers-only	No	Naturalistic urban cycling behaviour
**BikeZ-ETH-RA** Zürich, Switzerland	**6 Sep 2024**	**Yes**	**28**	**Drone (top-down)**	**Circular (10–14 m radius)**	**2.5–3.75 m**	**Bicycle-only**	**Yes**	**Free-flow, following, overtaking, and stop-and-go under varying densities and lane widths**

### Comparison with previous datasets

A variety of datasets related to cycling and vulnerable road users have been published in recent years, targeting applications ranging from object detection to trajectory prediction. However, these datasets differ substantially in terms of sensing modality, level of control, traffic context, and suitability for analysing bicycle traffic dynamics. Early controlled datasets such as the Wuppertal Experiment^33^ provide high-quality Cartesian trajectories and well-defined experimental conditions, but are limited in spatial extent, lane width, and behavioural diversity, focusing primarily on single-file following behaviour. In contrast, large-scale perception benchmarks such as the Tsinghua-Daimler Dataset^30^ and EuroCity Persons Dataset^31^ offer extensive image annotations across diverse urban environments, yet do not provide continuous trajectories or metric homography coordinate-transformations, restricting their applicability for traffic flow modelling and motion forecasting. The Stanford Drone Dataset^32^ introduced large-scale aerial observations and enabled multi-agent trajectory analysis across heterogeneous scenes. While influential, its mixed traffic composition, complex geometry, and scene-dependent coordinate projections make it difficult to isolate bicycle-bicycle interactions or study controlled traffic states. Similarly, datasets such as HNU^29^ and Xianxia-Jianhe-Shanghai^28^ provide cyclist trajectories in real-world traffic, but are recorded at intersections or shared spaces where interactions with motor vehicles, pedestrians, and infrastructure strongly influence behaviour. More recent datasets such as TUMDOT-MUC^47,48^ provide extensive naturalistic observations of two-wheelers over long recording durations. While highly valuable for studying real-world behaviour, these datasets are inherently uncontrolled, with heterogeneous traffic compositions, varying infrastructure, and discontinuous individual trajectories, limiting their use for systematic traffic flow experiments or controlled behavioural analysis. Table 2 compares the available bicycle trajectory datasets.

**Table 2:** Comparison of dedicated bicycle trajectory datasets.

Dataset	URL	Location	Track	Control	Mix. Modes	Frequency	Frames	Indivs.	Trajs.	Coords.	Description
Wuppertal Experiment^33^	Link	Wuppertal, Germany	Oval track (86 m)	Yes	No	25 Hz	—	5–33	Yes	Cartesian	Following behaviour, lane adherence
Tsinghua-Daimler Dataset^30^	Link	Beijing, China	Urban streets	No	Yes	—	14,674	—	No	Bound.Box	Cyclist and pedestrian detection
Stanford Drone Dataset^32^	Link	Stanford, USA	Campus scenes	No	Yes	30 Hz	~19,000	—	Partial	Bound.Box	Multi-agent trajectory analysis
EuroCity Persons Dataset^31^	Link	31 cities	Urban streets	No	Yes	—	47,300	—	No	Bound.Box	VRU detection benchmark
HNU Dataset^29^	Link	Hunan, China	Campus roads	No	Yes	25 Hz	5,678	—	Yes	Cartesian	Cyclist trajectory prediction
Xianxia-Jianhe-Shanghai Dataset^28^	Link	Shanghai, China	Urban intersection	No	Yes	8.33 Hz	—	680	Yes	Cartesian	Interaction-aware prediction
TUMDOT-MUC Dataset^47,48^	Link	Munich, Germany	Urban road (700 m)	No	No	12.5 Hz	~24,000	—	Yes	Cartesian	Bicycle traffic flow and interaction dynamics
**BikeZ-ETH-RA**	Link	Zürich, Switzerland	Circular closed-loop track	**Yes**	**No**	**25 Hz**	45,175	28	**Yes**	**Cartesian**	**Bicycle traffic flow and interaction dynamics**

The dataset presented here complements existing resources by providing a controlled, bicycle-only traffic environment with long, continuous trajectories recorded at high temporal resolution. The closed-loop circular track enables repeated interactions among the same cyclists and the observation of steady-state and transient traffic regimes, including stop-and-go waves, without confounding effects from mixed traffic or complex road geometry. In addition, the public availability of both raw aerial video and filtered Cartesian trajectories facilitates reproducibility, benchmarking, and reuse across traffic engineering, behavioural modelling, and data-driven trajectory prediction research. By bridging the gap between controlled laboratory-style experiments and uncontrolled naturalistic datasets, this dataset offers a unique empirical foundation for studying bicycle traffic flow dynamics and for developing and evaluating bicycle-aware models for safety-critical applications, including advanced driver assistance systems and autonomous driving.’

## Methods

### Experimental Setup

**Fig. 1:**
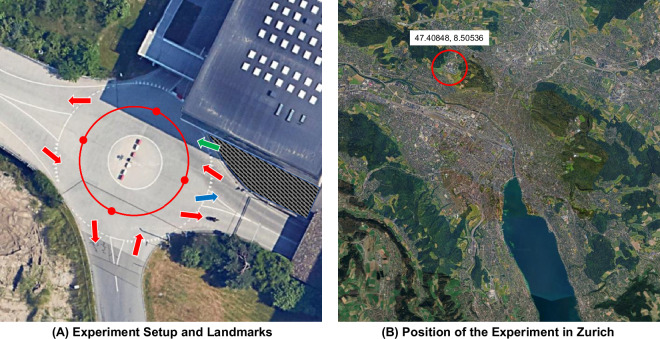
Study section. Aerial image provided from Google Maps.

**Fig. 2:**
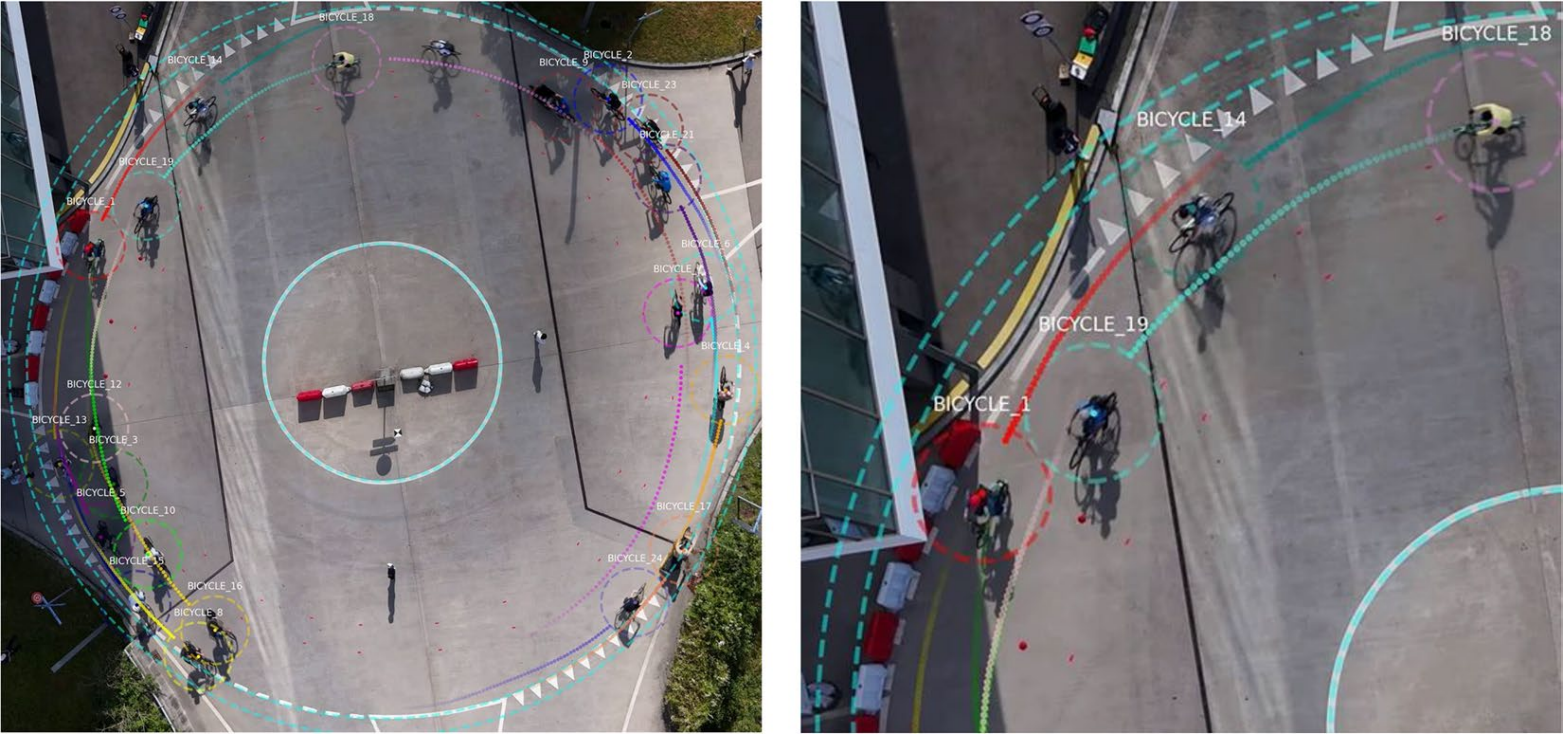
Trajectory dataset visualisation.

**Fig. 3:**
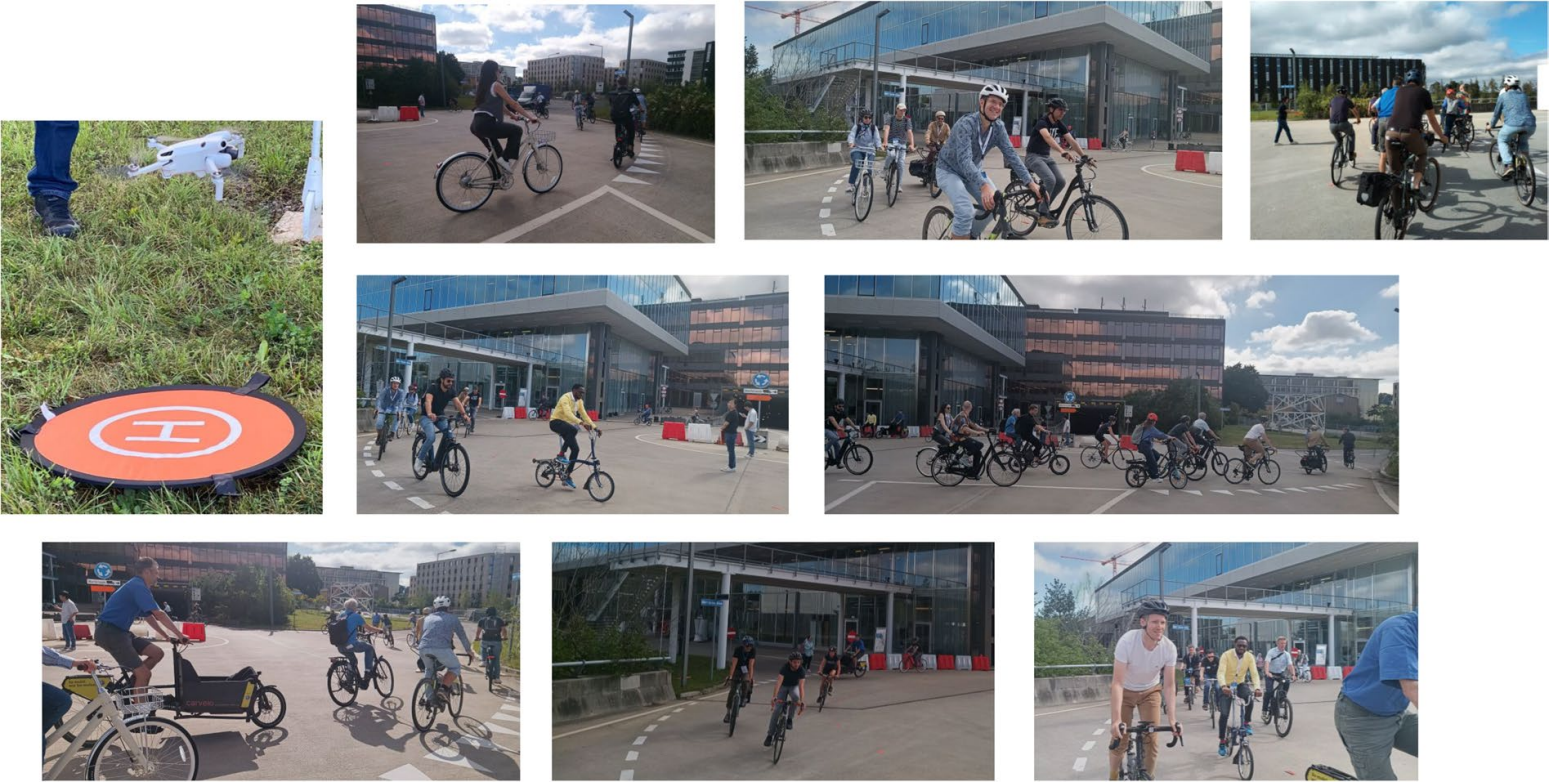
Impressions from the experiment. Participants provided informed written consent for video and image data to be published.

The controlled mass-cycling experiment was conducted near the Albert-Einstein-Garage at Hönggerberg Campus in ETH Zürich (47.40848^°^ N, 8.50536^°^ E; WGS84) on September 06th 2024, as shown in Figs. 1, 2 and 3. The number of bicycles was varied, and two lane widths were tested throughout different experiments. The traffic was frequently interrupted in a controlled manner to enable the observation of congestion and decongestion in the presence of numerous bicycles. The bicycles were provided by “Veloplan GmbH”, and participants for the experiment were recruited by the help of the “Cycling Research Board (CRB) Annual Meeting 2024”, and the public initiative “Pro Velo Kanton Zurich”. The bicycles included regular bicycles, e-bikes, and cargo bikes. The participants varied in their gender, age, and cultural background.

As humans have been included in the present study and experiments to generate this dataset, participants provided informed written consent for data sharing (both of video and photo material) before attending the experiment, and were made fully aware of any risks or implications of doing this. The collected data does not contain any sensitive locations or identifiable people. Only the photos from Fig. 3 might show identifiable people, that willingly provided their informed consent for doing so. The experiment was conducted in accordance with Helsinki Declaration and ETH Zurich Ethics Commission guidelines and received approval, which allowed for the data to be published under an open license.

Four objectives were considered when designing the experiment: (i) to observe mass-cycling traffic dynamics in a controlled setup, (ii) to record very long bicycle trajectories, (iii) to produce the fundamental diagram (FD) of bicycle flow and collect behavioural parameters to calibrate microscopic simulation models, and (iv) to observe interactions between bicyclists without effects of road geometry or mixed traffic.

Moreover, the controlled experiment was conducted in four different stages, as summarised below:

- **Stage 1: Free-flow state with a wide lane width (2.5 m)**. We started with 6 cyclists, and added 3 cyclists every minute. With the gradual increment of the number of cyclists, we were able to produce the undersaturated, free-flow traffic states on the fundamental diagram. Some small disturbances occurred near the on-ramp when the density was large enough, creating congested states.
- **Stage 2: Stop-and-go traffic with a wide lane width (2.5 m)**. In this stage, we aimed to allow everyone from stage 1 to stay in the ring and continued with adding further participants. Every two minutes, one helper served as a virtual traffic light to stop the flow in the ring for a while to create disturbances and backward-propagating waves. The stopping time lengths varied between 10 s, 20 s, and 30 s to ensure that with a different number of participants all came to a stop before the helper switched to green again. This enabled the observation of severely congested states.
- **Stage 3: Free-flow state with a wide lane width (3.75 m)**. We repeated stage 1, but at a wider lane width.
- **Stage 4: Stop-and-go traffic with a wide lane width (3.75 m)**. We repeated stage 2, but at a wider lane width.

Figure 1 illustrates the spatial experiment setup (A) and the location of the experiment in the Zurich region (B). The setup in (A) contains red arrows that represent possible entries for cars and trucks that may disturb the experiment. The red dots represent the helpers that stand on the inner ring and coordinate the experiment. The cyclists ride between the helpers and the outer ring. When cars or trucks approach, helpers are advised to stop the cyclists so that the motorised vehicles can pass. Bicyclists can enter the ring from one on-ramp (green arrow) and leave the ring from one off-ramp (blue arrow). Resting participants wait in the designated waiting zone (black area).

The source code that was used to optically extract trajectories from video files and process them (via Kalman filtering) can be found in the dataset’s GitHub repository online: https://github.com/DerKevinRiehl/mass_cycling_experiment.

### Optical Trajectory Extraction

**Fig. 4:**
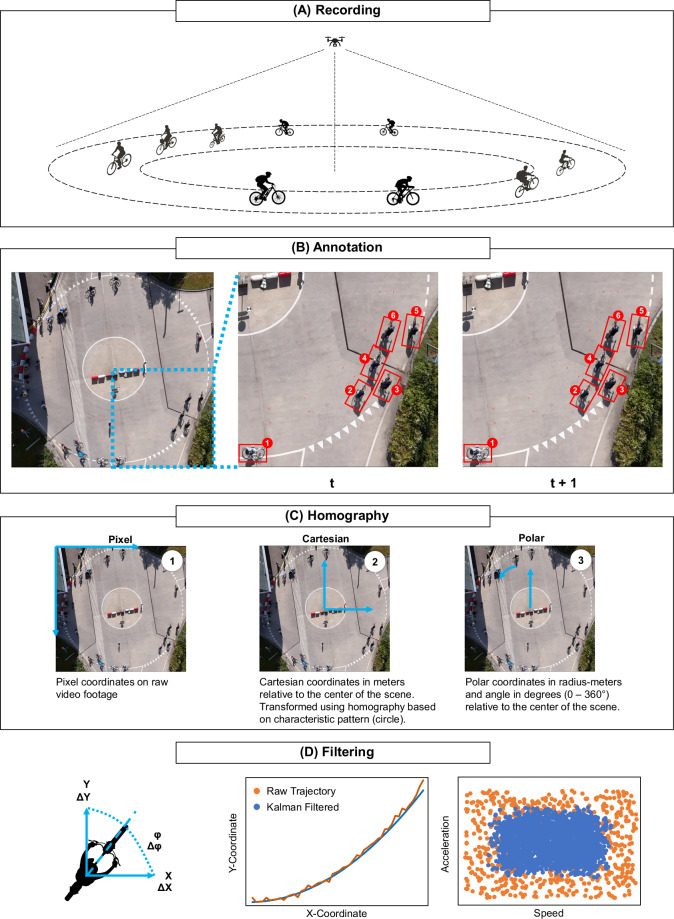
Optical Trajectory Extraction & Filtering.

The cyclists were recorded with a drone from an aerial, top-down perspective throughout the experiment, from a height of 50m above ground, as shown in Fig. 4(A). The drone model *DJI Mini 4 Pro* was used, and the generated video material has a resolution of 3840 x 2160 pixels, at a frame rate of 25 FPS. The total recording comprises 30 minutes of video material (25.7 GB video data) in 9 separate files.

In each frame of the videos, all cyclists were annotated using oriented object detection models *YOLOv8x*^50^ (https://yolov8.com/) which were complemented by manual annotation, following the methodology described in^46^. Consecutively, the oriented bounding box annotations were matched from frame to frame to unique bicycle IDs to enable tracking across time, using the frame-to-frame-matching method^46^ that was complemented with manual matching. This was the most labour-intensive step, as it required significant manual effort to ensure quality control and is summarised in Fig. 4(B).

The resulting, unfiltered bicycle trajectories were transformed from raw pixel coordinates to a Cartesian, metric coordinate system, where the centre of the circular track is the centre of the coordinate system, as described in Fig. 4(C). For this purpose, the position of the markings of the circular track on the road was extracted using *Circular Hough Transform*^51^. Given the known geometry of the circles diameter *D*^*cart*^ and its diameter *D*^*px*^ and position on the image with pixel coordinates ${\overrightarrow{P}}_{C}^{px}$, annotation coordinates in pixel coordinates ${\overrightarrow{P}}_{A}^{px}$ could be transformed to Cartesian coordinates ${\overrightarrow{P}}_{A}^{cart}$ (relative to the circle) as follows:

1 $${\overrightarrow{P}}_{A}^{cart}=\frac{{D}^{cart}}{{D}^{px}}({\overrightarrow{P}}_{A}^{px}-{\overrightarrow{P}}_{C}^{px})$$

Furthermore, the coordinates were expressed in polar coordinates ${\overrightarrow{P}}_{A}^{pol}$ as a vector consisting of two components ${r}_{A}^{pol}$ and ${\theta }_{A}^{pol}$:

2 $${r}_{A}^{pol}=\left\Vert {\overrightarrow{P}}_{A}^{cart}\right\Vert \,\,\,\,\,\,\,\,{\rm{and}}\,\,\,\,\,\,\,\,{\theta }_{A}^{pol}=arctan2({\overrightarrow{P}}_{A}^{cart})$$

The coordinates in polar coordinates allow for the calculation of metric lane coordinates ${\overrightarrow{P}}_{A}^{lan}$ as a vector of two components ${x}_{A}^{lan}$ and ${y}_{A}^{lan}$ that reflect lateral offset from the lane centre, and progress along the (infinitely long) lane, where ${\widetilde{\theta }}_{A}^{pol}$ is a monotonically increasing angle for every completed round on the circle that is calculated using an isotonic regression:

3 $${x}_{A}^{lan}={r}_{A}^{pol}-\frac{{D}^{cart}}{2}\,\,\,\,\,\,\,\,\,{\rm{and}}\,\,\,\,\,\,\,\,\,{y}_{A}^{lan}=\frac{{D}^{cart}}{2}{\widetilde{\theta }}_{A}^{pol}$$

The lane coordinates can be used for trajectory forecasting, lane-changing, and bicycle-following models.

### Physically-Plausible Trajectory Filtering

The raw, Cartesian trajectory was filtered with a physically-plausible, extended Rauch-Tung-Striebel, Kalman-filtering approach^46^ as outlined in the following and showcased in Fig. 4(D). The bicycle state is reflected in its Cartesian position (*x*, *y*), its velocities (*v*_*x*_ = *δx*/*δt*, *v*_*y*_ = *δy*/*δt*), the orientation (direction) (*φ*) and angular velocity (*ω* = *δφ*/*δt*). The following kinematic model describes the relationship between the bicycle states and was used for the extended Kalman filter^52,53^, where *Δt* represents the simulation time step (*Δt* = 40 ms at 25 Hz):

4 $$X[k+1]=f(X[k])=\left(\begin{array}{c}x+cos(\alpha )v\Delta t\\ y+sin(\alpha )v\Delta t\\ \alpha +\omega \Delta t\\ v\\ \omega \end{array}\right)$$

The extended Kalman filtering was conducted both forward and backward in time and the average of the two was taken, following Rauch-Tung-Striebel interval smoothing^46,54^.

## Data Records

The dataset (trajectory data and videos) is available at Zenodo^55^ https://zenodo.org/records/18098714. It comprises nine video recordings as shown in Table 3 which were separated into 21 sequences, as summarised in Table 4. For each frame (time step) of the sequences, the trajectories of all riding bicycles are provided with all parameters shown in Table 5.

**Table 3:** Aerial video recording file summary.

	Video File Name	Timestamp	Duration	File Size	Lane Width
Stage 1	DJI_20240906103036_0003_D.MP4	10:30:36	04:06	3.50 GB	2.50 m
	DJI_20240906103442_0004_D.MP4	10:34:42	04:07	3.50 GB	2.50 m
	DJI_20240906103850_0005_D.MP4	10:38:50	04:08	3.50 GB	2.50 m
Stage 2	DJI_20240906104511_0007_D.MP4	10:45:11	04:05	3.50 GB	2.50 m
	DJI_20240906104917_0008_D.MP4	10:49:17	04:04	3.50 GB	2.50 m
	DJI_20240906105321_0009_D.MP4	10:53:21	00:56	0.836 GB	2.50 m
Stage 3	DJI_20240906105621_0010_D.MP4	10:56:21	04:05	3.50 GB	3.75 m
Stage 4	DJI_20240906110027_0011_D.MP4	11:00:27	04:04	3.50 GB	3.75 m
	DJI_20240906110432_0012_D.MP4	11:04:32	00:28	0.426 GB	3.75 m
	**Total**		**30:07**	**25.7 GB**

**Table 4:** Video sequences summary with frame and bicycle counts.

	Sequence Nr.	Video file name	Sequence name	From (frame)	To(frame)	# Frames	# Bicycles
Stage 1	1	DJI_20240906103036_0003_D.MP4	PART_1	300	1950	1650	6
	2	DJI_20240906103036_0003_D.MP4	PART_2X	2425	3725	1300	10
	3	DJI_20240906103036_0003_D.MP4	PART_3X	4800	5350	550	10
	4	DJI_20240906103036_0003_D.MP4	PART_4	5625	6154	529	14
	5	DJI_20240906103442_0004_D.MP4	PfART_1X	0	1700	1700	14
	6	DJI_20240906103442_0004_D.MP4	PART_2X	2850	5750	2900	19
	7	DJI_20240906103850_0005_D.MP4	PART_1X	325	2500	2175	22
Stage 2	8	DJI_20240906104511_0007_D.MP4	PART_1X	200	2800	1675	20
	9	DJI_20240906104511_0007_D.MP4	PART_23X	3000	6128	3128	20
	10	DJI_20240906104917_0008_D.MP4	PART_1X	1475	3775	2300	19
	11	DJI_20240906104917_0008_D.MP4	PART_2X	4100	5500	1400	20
	12	DJI_20240906105321_0009_D.MP4	PART_1	150	350	200	13
Stage 3	13	DJI_20240906105621_0010_D.MP4	PART_1	350	925	575	6
	14	DJI_20240906105621_0010_D.MP4	PART_2	1250	1900	650	9
	15	DJI_20240906105621_0010_D.MP4	PART_3	2250	2875	625	12
	16	DJI_20240906105621_0010_D.MP4	PART_4	3075	3250	175	16
Stage 4	17	DJI_20240906105621_0010_D.MP4	PART_5X	3250	4050	800	17
	18	DJI_20240906105621_0010_D.MP4	PART_6X	5750	6138	388	17
	19	DJI_20240906110027_0011_D.MP4	PART_1X	0	2175	2175	17
	20	DJI_20240906110027_0011_D.MP4	PART_2345X	2275	6122	3847	17
	21	DJI_20240906110432_0012_D.MP4	PART_1	0	625	625	17
**Total**					**29367**	**21**

**Table 5:** Trajectory dataset column summary.

Parameter	Description	Example Value	Unit
Vehicle_ID	String ID of the bicycle	BICYCLE_18	[-]
Frame_ID	Numeric frame ID (i.e. number)	3	[-]
GlobalTime	Time from the start of the video	0.12	[s]
Cartesian_X	Cartesian X position of bicycle	−16.58	[m]
Cartesian_Y	Cartesian Y position of bicycle	25.24	[m]
Polar_X	Polar coordinate X position (radius)	4.84	[m]
Polar_Y	Polar coordinate Y position (angle)	14.74	[rad]
v_Length	Length of the bicycle	1.8	[m]
v_Width	Width of the bicycle	0.64	[m]
v_Vel	Longitudinal speed of the bicycle	1.85	[m/s]
v_Angle	Directional orientation of the bicycle	3.14	[rad]
v_AngleVel	Angular speed of the bicycle	0.02	[rad/s]

The video recordings were split into the sequences given a different number of bicycles, disruptions (e.g., drone landing for battery, controlled traffic flow disturbance, or crossing of motorised vehicles). A detailed description of the shown scenes for different points in time is provided in Table 6. Note that the total number of frames in these sequences (29,367) is lower than the theoretical maximum for 30 minutes at 25 Hz (45,000) because frames corresponding to drone landings, temporary interruptions due to passing motorised vehicles, or brief occlusions were excluded to ensure data quality and consistency within each sequence.

**Table 6:** Video sequence descriptions.

	Video file name	From	To	# Bicycles	Description
Stage 1	DJI_20240906103036_0003_D.MP4	00:12	01:18	6	6 bikes riding
	DJI_20240906103036_0003_D.MP4	01:18	01:37		Bikes joining
	DJI_20240906103036_0003_D.MP4	01:37	02:18	10	10 bikes riding
	DJI_20240906103036_0003_D.MP4	02:18	03:28		Bikes stop, motorised vehicles cross
	DJI_20240906103036_0003_D.MP4	03:28	03:34	10	10 bikes riding
	DJI_20240906103036_0003_D.MP4	03:34	03:45		Bikes joining
	DJI_20240906103036_0003_D.MP4	03:45	end	14	14 bikes riding
	DJI_20240906103442_0004_D.MP4	00:00	00:55	14	14 bikes riding
	DJI_20240906103442_0004_D.MP4	00:55	01:38		Bikes stop, motorised vehicles cross
	DJI_20240906103442_0004_D.MP4	01:38	01:54		Bikes joining
	DJI_20240906103442_0004_D.MP4	01:54	03:00	18	18 bikes riding
	DJI_20240906103442_0004_D.MP4	03:00	end		Bikes stop, motorised vehicles cross
	DJI_20240906103850_0005_D.MP4	00:13	01:22	22	22 bikes riding
	DJI_20240906103850_0005_D.MP4	01:22	02:16		Bikes stop, motorised vehicles cross
	DJI_20240906103850_0005_D.MP4	02:16	end		Drone initiates landing procedure
Stage 2	DJI_20240906104511_0007_D.MP4	00:00	00:18		Bikes stop, motorised vehicles cross
	DJI_20240906104511_0007_D.MP4	00:18	01:25	20	20 bikes riding
	DJI_20240906104511_0007_D.MP4	01:25	02:17		Bikes stop, motorised vehicles cross
	DJI_20240906104511_0007_D.MP4	02:17	03:30	20	20 bikes riding
	DJI_20240906104511_0007_D.MP4	03:30	end	20	Controlled traffic flow disturbance
	DJI_20240906104917_0008_D.MP4	00:00	01:11		Bikes stop, motorised vehicles cross
	DJI_20240906104917_0008_D.MP4	01:11	02:11	19	19 bikes riding
	DJI_20240906104917_0008_D.MP4	02:11	02:52		Bikes stop, motorised vehicles cross
	DJI_20240906104917_0008_D.MP4	02:52	03:28	20	20 bikes riding
	DJI_20240906104917_0008_D.MP4	03:28	end		Bikes stop, motorised vehicles cross
	DJI_20240906105321_0009_D.MP4	00:00	00:06		Bikes stop, motorised vehicles cross
	DJI_20240906105321_0009_D.MP4	00:06	end	13	13 bikes leaving
Stage 3	DJI_20240906105621_0010_D.MP4	00:00	00:14		Bikes entering
	DJI_20240906105621_0010_D.MP4	00:14	00:37	6	6 bikes riding
	DJI_20240906105621_0010_D.MP4	00:37	00:50		Bikes joining
	DJI_20240906105621_0010_D.MP4	00:50	01:16	9	9 bikes riding
	DJI_20240906105621_0010_D.MP4	01:16	01:30		Bikes joining
	DJI_20240906105621_0010_D.MP4	01:30	01:55	12	12 bikes riding
	DJI_20240906105621_0010_D.MP4	01:55	02:03		Bikes joining
	DJI_20240906105621_0010_D.MP4	02:03	02:10	16	16 bikes riding
	DJI_20240906105621_0010_D.MP4	02:10	02:28	17	17 bikes riding
	DJI_20240906105621_0010_D.MP4	02:28	03:58		Bikes stop, motorised vehicles cross
	DJI_20240906105621_0010_D.MP4	03:58	end	17	17 bikes riding
Stage 4	DJI_20240906110027_0011_D.MP4	00:00	01:09	17	17 bikes riding
	DJI_20240906110027_0011_D.MP4	01:09	01:41		Bikes stop, motorised vehicles cross
	DJI_20240906110027_0011_D.MP4	01:41	02:12	17	17 bikes riding
	DJI_20240906110027_0011_D.MP4	02:12	02:20		Controlled traffic flow disturbance
	DJI_20240906110027_0011_D.MP4	02:20	02:55	17	17 bikes riding
	DJI_20240906110027_0011_D.MP4	02:55	03:07		Controlled traffic flow disturbance
	DJI_20240906110027_0011_D.MP4	03:07	03:40	17	17 bikes riding
	DJI_20240906110027_0011_D.MP4	03:40	03:54		Controlled traffic flow disturbance
	DJI_20240906110027_0011_D.MP4	03:54	end	17	17 bikes riding
	DJI_20240906110432_0012_D.MP4	00:00	00:25	17	17 bikes riding
	DJI_20240906110432_0012_D.MP4	00:25	end		Bikes leaving

## Usage Notes

We acknowledge that the dataset is based on a relatively small sample of 28 participants in a single experiment conducted at one location. While this controlled setting provides high-resolution, consistent trajectories and a unique opportunity to study bicycle traffic dynamics without confounding factors, the limited number of participants and the single-track environment may constrain the generalizability of findings to broader, more heterogeneous populations or complex real-world traffic settings. Furthermore, although the dataset captures realistic bicycle interactions, it was collected in a controlled environment involving helpers, artificial flow interruptions, and a closed-loop circular track. These design elements, while enabling systematic observation of phenomena such as stop-and-go waves and steady-state behaviours, mean that the setting can only partially be considered “fully naturalistic”. Future studies expanding the number of participants, including multiple track designs or naturalistic urban environments, would help validate and extend the insights derived from this dataset. Nevertheless, despite the sample size, the dataset offers valuable empirical observations of fundamental bicycle traffic behaviours, including stop-and-go waves and steady-state dynamics, which are often difficult to isolate in naturalistic traffic data.

Although the total number of participants (28 cyclists) may appear modest compared to large-scale crowd experiments, the term “mass-cycling” in this context refers to the collective occupation of space and the emergence of traffic flow dynamics rather than the absolute number of individuals. For the circular test track geometry and lane widths considered, 28 cyclists were sufficient to span a wide range of density states, from free-flow to stop-and-go regimes, enabling the construction of the complete fundamental diagram (flow-density-speed relationship) and the analysis of collective cycling dynamics. Thus, “mass” refers to the system-level traffic behaviour emerging from spatial interaction, not merely to participant count.

## Technical Validation

In this section, we validate the dataset from four perspectives. First, we assess the quality and plausibility of the trajectories using frame-to-frame noise levels, and speed-acceleration profiles. Second, the trajectory extraction is supported by manual annotations collected at 0.5 Hz, providing a self-contained reference for evaluating the accuracy of the extracted bicycle trajectories. We report statistical error metrics by comparing the filtered trajectories against these manual annotations. Third, we examine the quality and realism of the dataset by analysing bicycle-bicycle interactions and patterns of lateral space utilisation. Fourth, we assess the relationships between lateral space utilisation, cycling speed, and the lane-width configurations permitted at the time of experiment conduction.

**Fig. 5:**
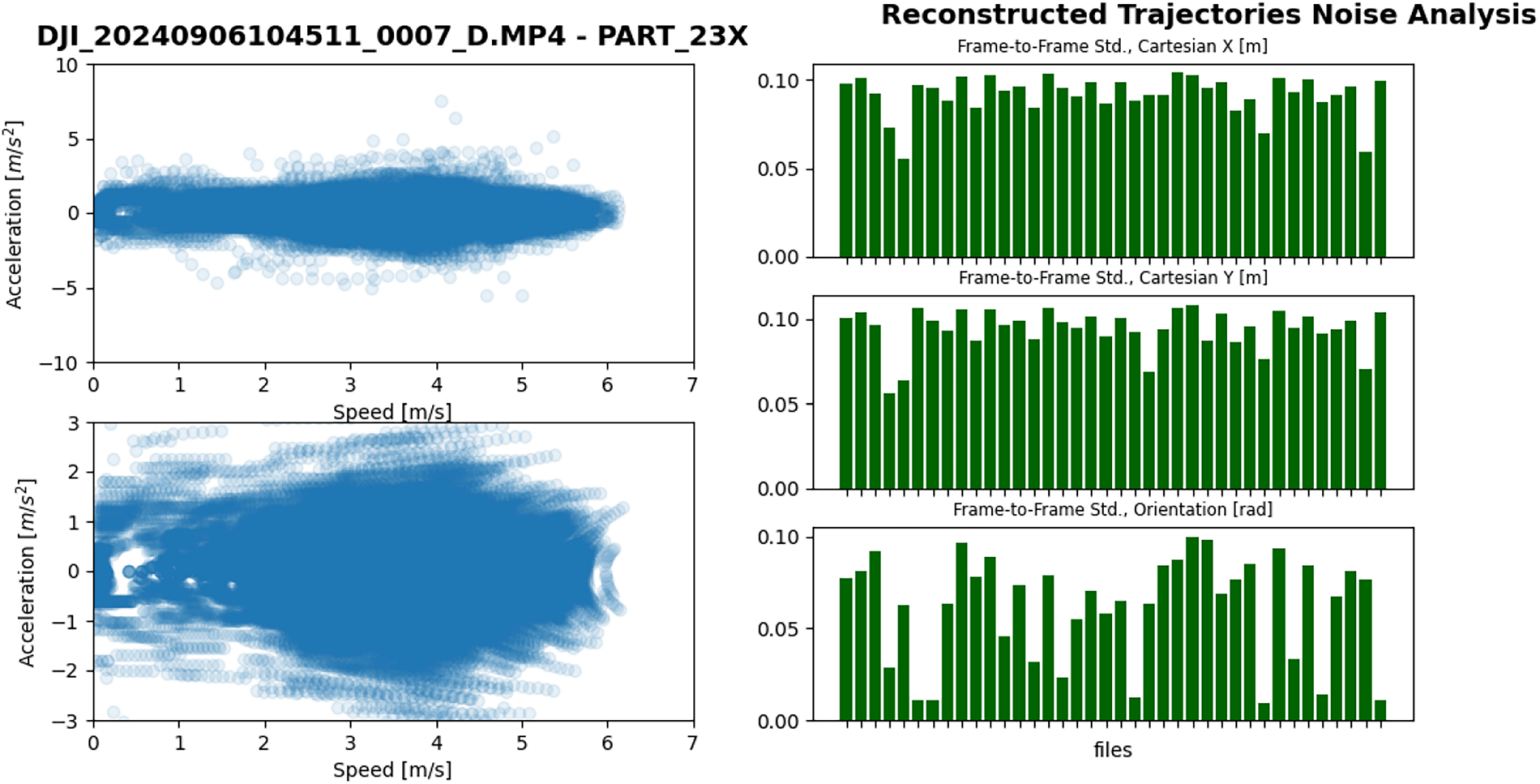
Physical plausibility and noise analysis.

Regarding physical plausibility, the acceleration profiles for given speeds are displayed in the left plots of Fig. 5 for the longest video sequence DJI_20240906104511_0007_D.MP4 (PART_23X). The trajectories demonstrate consistent and physically-plausible acceleration profiles, with more than 98.76% of all observations between −2 and 2 *m*/*s*^2^ – accelerations that are plausible for bicycle kinematics^56^. An analysis of the positional and orientational noise levels (from one to the next frame) across all sequences further supports the plausibility of the dataset, and can be found in the right plots of Fig. 5. Positional noise levels of less than 10 cm and directional noise levels of less than 0.10 rad (5.73^°^) were observed.

**Fig. 6:**
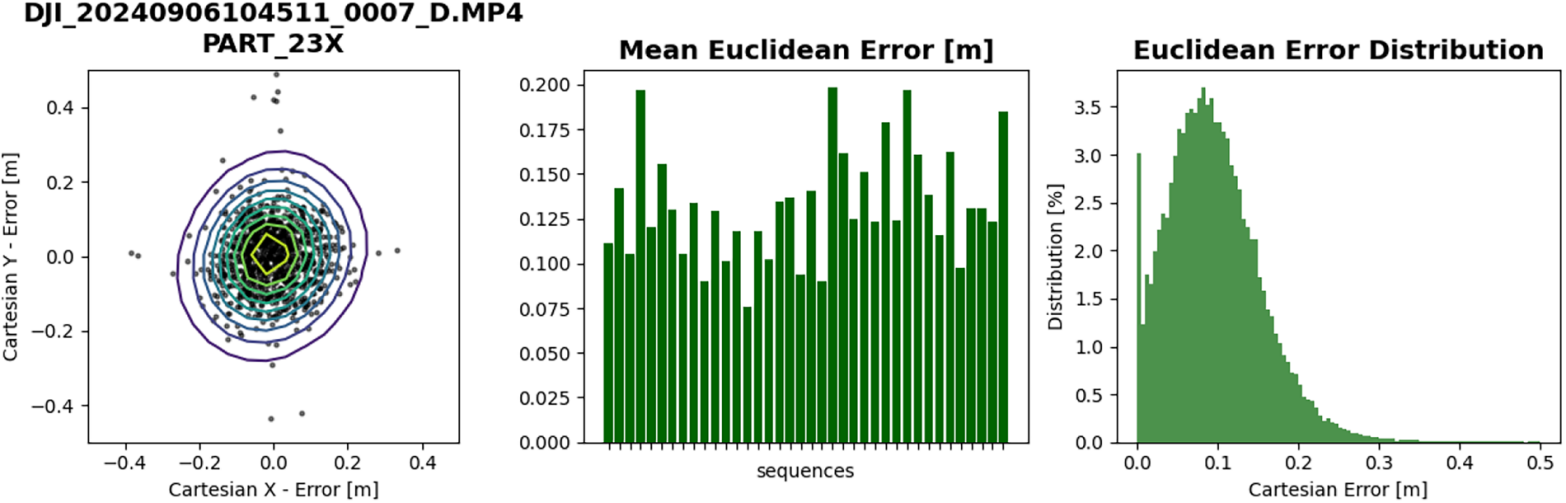
Error analysis. Figure shows manual annotation vs. filtered trajectory.

Regarding deviations from manual annotations, we examined the error distribution of the filtered trajectories relative to the manually annotated reference positions (sampled at 0.5 Hz), as presented in Fig. 6. The rightmost plot illustrates the distribution of spatial deviations for the longest video sequence (DJI_20240906104511_0007_D.MP4, PART_23X). The results indicate that more than 95% of all observations exhibit deviations smaller than 0.15 m, and the nearly isotropic distribution of errors suggests the absence of systematic bias or directional filtering artefacts. The central plot shows the distribution of mean Euclidean errors across all sequences, all of which remain below 0.20 m. Finally, the rightmost plot, summarising the Euclidean error distribution across all datasets, demonstrates that over 99% of all observations have Euclidean errors below 0.20 m. While no standard benchmark exists for bicycle trajectory extraction from aerial video, these error levels are consistent with or better than those reported in previous studies of video-based pedestrian and vehicle tracking^18,32,45,46^. Accordingly, the filtered trajectories can be considered to exhibit high precision and reliability for downstream analyses, although some extreme errors may occur in brief periods of occlusion or temporary interruptions (see right tail of Euclidean error distribution in Fig. 6). These results collectively confirm the high precision and consistency of the derived trajectories, thus indicating excellent data quality.

**Fig. 7:**
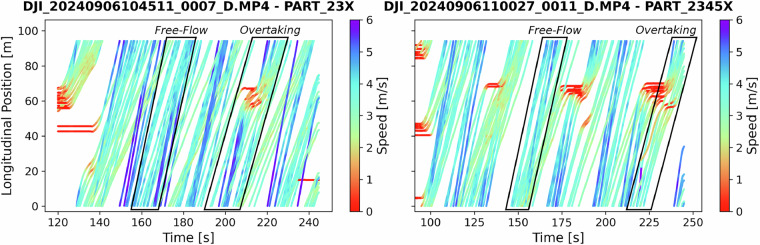
Time-space diagrams.

Regarding bicycle-bicycle interactions, Fig. 7 presents time-space diagrams of the cyclists’ longitudinal motion, with the circular track unwrapped into a 94-m straight roadway for visualisation. For illustration, we use the two longest video sequences: DJI_20240906104511_0007_D.MP4 (PART_23X) with 3,128 frames, and DJI_20240906110027_0011_D.MP4 (PART_2345X) with 3,847 frames. During unperturbed periods of the experiment, highlighted as “Free-Flow” regions in Fig. 7, the cyclists maintain comparable average speeds, and the longitudinal trajectories appear largely parallel. This indicates minimal overtaking activity and suggests that cyclists travel at their preferred speeds without major interactions. In contrast, during periods where controlled disturbances were introduced into the flow, overtaking increases, as reflected by intersecting trajectories (see “Overtaking” regions in Fig. 7). Overall, in the absence of external perturbations, any potential build-up of longitudinal congestion tends to be “absorbed” laterally: faster cyclists overtake without the need for explicit lane-changing rules, unlike lane-based motorised vehicular traffic. This behaviour highlights the distinct interaction mechanisms of cycling traffic and its non-lane-based nature.

**Fig. 8:**
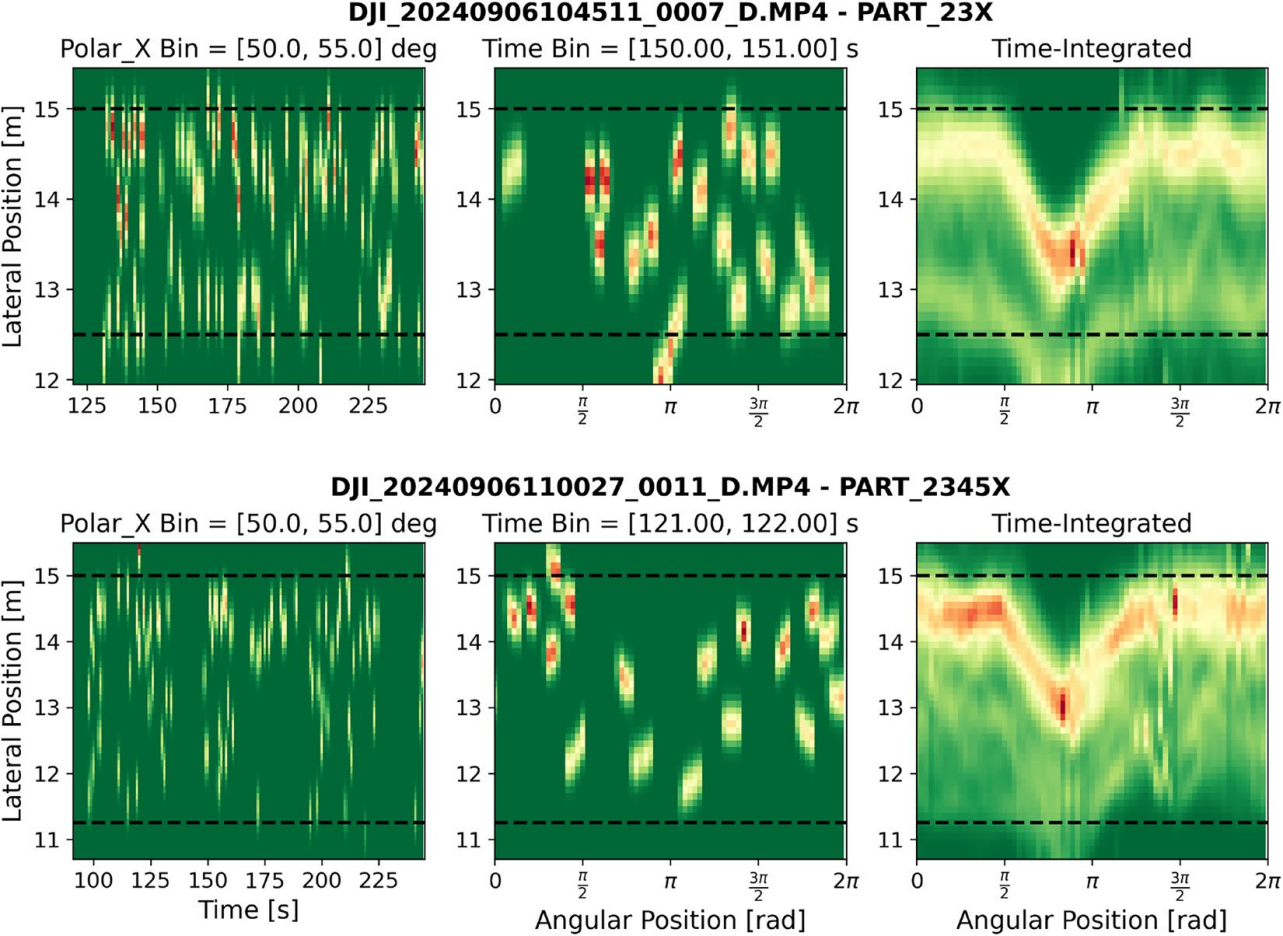
Lateral spatial distribution. Figure shows green to red colour scale with red representing the highest probability density.

**Fig. 9:**
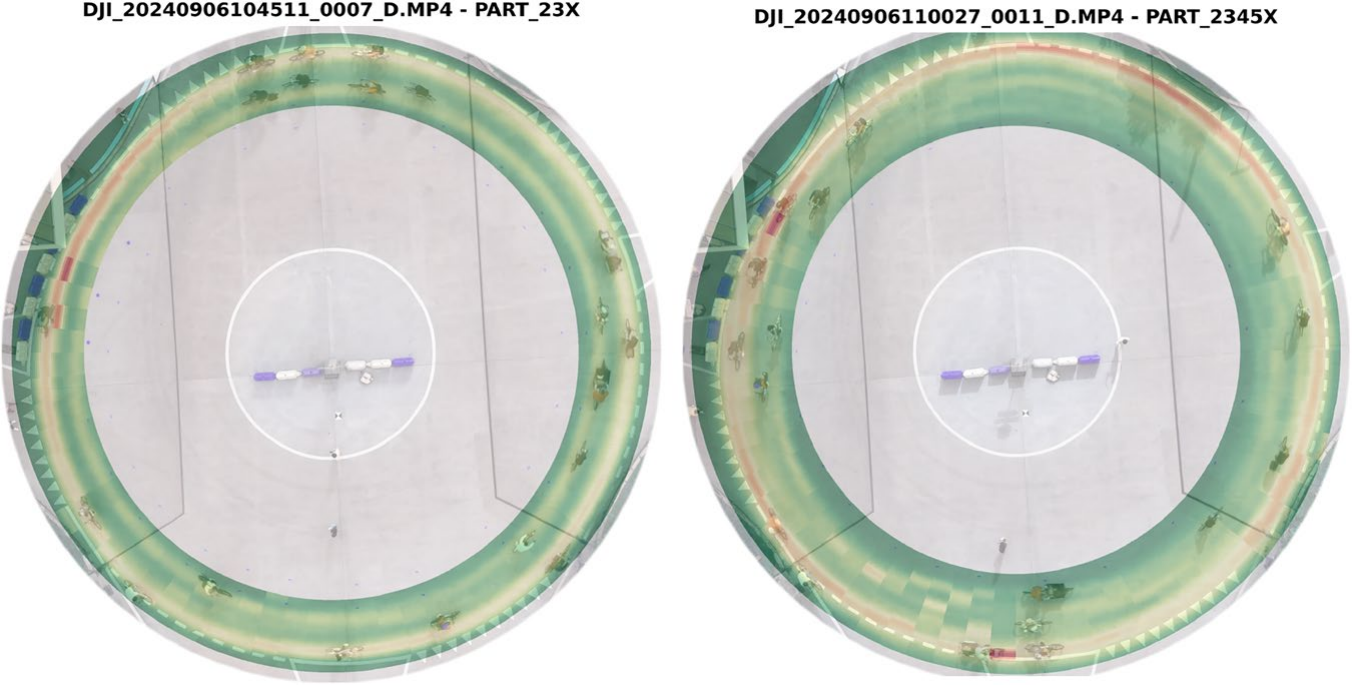
Lateral spatial distribution. Figure shows map view, and green to red colour scale with red representing the highest probability density.

Figure 8 illustrates the lateral space utilisation for the same two video sequences discussed previously. The black dashed lines represent the imposed lane-width configurations and their corresponding boundaries. Lateral utilisation is quantified through a probability density function (PDF), ${\mathbb{P}}({\theta }_{A}^{P},{r}_{A}^{P},t)$, which encodes the likelihood of observing a cyclist at polar position $({\theta }_{A}^{P},{r}_{A}^{P})$ at time *t*. To account for the finite bicycle width, *w*_*b*_, this PDF is convolved with a 3D Gaussian kernel such that 3*σ*_*r*_ = *w*_*b*_, where *σ*_*r*_ is the standard deviation of the filter in the radial direction. We exemplify the resulting PDF in three ways: (a) at a fixed polar-angle bin of ${\theta }_{A}^{P}\in {[50,55]}^{\circ }$, (b) at a fixed time interval of *t* ∈ [150, 151] s, and (c) integrated over time as ${\mathbb{P}}({\theta }_{A}^{P},{r}_{A}^{P})$ to provide an overall depiction of lateral usage within each video sequence. Finally, the time-integrated PDF is visualised in map view in Fig. 9. As shown in Figs. 8 and 9, cyclists demonstrate a clear preference for riding closer to the outer lane boundary, with a substantially lower probability density observed near the inner boundary. Occasional boundary violations occur, but with low likelihood. This behavioural trend remains consistent across both lane-width configurations and participant densities (20 cyclists with a 2.5-m lane width, and 17 cyclists with a 3.75-m lane width). The downward dip in the lateral position around the angular position of ${\theta }_{A}^{P}\approx \pi$ (rad) is due to the actual track configuration. The track is not a perfect circle and is constrained by the surrounding buildings, as seen in the top left corner of Fig. 2.

**Fig. 10:**
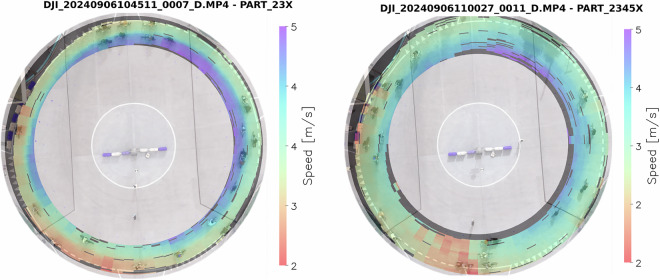
Lateral spatial distribution versus speed. Figure shows map view.

Finally, the relationship between lateral space utilisation, cycling speed, and the permitted lane width is examined in Fig. 10. Following the same procedure as earlier, we estimate the conditional probability density ${\mathbb{P}}(v| {\theta }_{A}^{P},{r}_{A}^{P},t)$ and integrate it over time to obtain ${\mathbb{P}}(v| {\theta }_{A}^{P},{r}_{A}^{P})$. Figure 10 visualises the expected cycling speed, ${\mathbb{E}}[v| {\theta }_{A}^{P},{r}_{A}^{P}]$, as a function of polar position $({\theta }_{A}^{P},{r}_{A}^{P})$. The results indicate that faster cyclists tend to travel closer to the inner boundary (i.e., overtaking from the left), while slower cyclists gravitate toward the outer boundary. This spatial-speed stratification highlights a key mechanism of “congestion absorption” in bicycle traffic: due to the lane-free nature of the flow and the self-organizing behaviour of cyclists, speed differences are accommodated laterally rather than resulting in longitudinal queuing. For the video sequence DJI_20240906110027_0011_D.MP4 (PART_2345X), a controlled stop disturbance was enforced between approximately *t* ∈ [210, 225] s at angular position ${\theta }_{A}^{P}\approx \frac{3\pi }{2}$ rad (see Figs. 7, 8, and 10). Although this disturbance did not fully saturate the available lateral space, it triggered numerous overtaking manoeuvres. Consequently, the expected speed distributions near the disturbance deviate from the broader spatial pattern, as cyclists actively reorganise laterally to mitigate its impact.

### Ethics statement

Human participants have been included in the present study and experiments to generate this dataset. We confirm that there are no images of any identifiable people or sensitive locations shown in the video dataset. We confirm that there are no images of sensitive locations shown in the manuscript. We confirm that there are images of identifiable people in the manuscript. Participants provided informed written consent for video and image data to be published, and furthermore provided informed consent for their identities (in form of pictures in the manuscript) to be shared, and were made fully aware of any risks or implications of doing this. The Helsinki Declaration standards were applied during experiment conduct. The experiment was conducted in accordance with ETH Zurich Ethics Commission guidelines and received approval, which allowed for the data to be published under an open license.

## Data Availability

The video files andfiltered trajectory data for all sequences can be found on Zenodo: 10.5281/zenodo.18098714. Furthermore, three demonstration videos can be found on YouTube: https://youtu.be/-UbFwMcpJNk, and https://youtu.be/b4SxqPf-qpQ, and https://youtu.be/mCQmYtJ3D8w. More details can be found on the project site https://www.bikez.ethz.ch/.

## Code availability

The source code that was used to optically extract trajectories from video files and process them (via Kalman filtering) can be found in the dataset’s GitHub repository online: https://github.com/DerKevinRiehl/mass_cycling_experiment.
